# Predicting the early risk of ophthalmopathy in Graves’ disease patients using TCR repertoire

**DOI:** 10.1002/ctm2.218

**Published:** 2020-11-04

**Authors:** Yue Wang, Yufeng Liu, Xiaofei Yang, Hui Guo, Jiadong Lin, Jinkui Yang, Mingqian He, Jingya Wang, Xiaomei Liu, Tingting Shi, Liping Wu, Chengsheng Zhang, Kai Ye, Bingyin Shi

**Affiliations:** ^1^ Department of Endocrinology The First Affiliated Hospital of Xi'an Jiaotong University Xi'an China; ^2^ MOE Key Lab for Intelligent Networks and Networks Security Faculty of Electronic and Information Engineering Xi'an Jiaotong University Xi'an China; ^3^ Precision Medicine Center The First Affiliated Hospital of Xi'an Jiaotong University Xi'an China; ^4^ Genome institute The First Affiliated Hospital of Xi'an Jiaotong University Xi'an China; ^5^ BioBank The First Affiliated Hospital of Xi'an Jiaotong University Xi'an China; ^6^ School of Computer Science and Technology Faculty of Electronic and Information Engineering Xi'an Jiaotong University Xi'an China; ^7^ Department of Endocrinology Beijing Tongren Hospital Capital Medical University Beijing China; ^8^ Beijing Key Laboratory of Diabetes Research and Care Beijing China; ^9^ Department of Endocrinology Nanjing First Hospital Nanjing Medical University Nanjing China; ^10^ The Jackson Laboratory for Genomic Medicine Farmington Connecticut

To the Editor:

At present, predicting the early risk of ophthalmopathy in Graves’ disease patients is extremely difficult. In this study, we proposed a novel score—TCR clonal expansion and chaos score (TCS)—to characterize TCR V‐J combination (VJ). And VJ signatures that could distinguish Graves’ ophthalmopathy (GO) from Graves’ hyperthyroidism (GH) were used to successfully predict GO progression.

GH is an organ‐specific autoimmune disease, with an annual incidence of 20‐50 cases per 100 000 persons.^1^ GO characterized by disfiguring and dysfunctioning features of corneal breakdown or optic neuropathy occurs in 3‐5% GH patients, which significantly decreases the quality of life.^1^ Identification of signatures that prospectively predict progression from GH to GO may open the possibility for prophylactic intervention to prevent GO. Two studies have reported that restricted usage of TCR may contribute to the recruitment and oligoclonal expansion of T cells in early stage of GO.^2^, ^3^ As GO is autoimmune‐mediated inflammation and TCR repertoires snapshot current accumulated adaptive immune events, we reasoned that specific TCR signatures emerging before ophthalmopathy presentation might be appropriate predictors for GO progression.

It was reported that T cells showing clonal expansion in tissues such as kidney or tumors can be traced in blood, enabling monitor of disease progression.^4^, ^5^ In this study, we first compared T‐cell repertoires between thyroids and blood from six GH patients, and confirmed that the majority of expanded clonotypes (with frequencies over 0.001) in thyroids could be detected in blood (Figure 1A,B). Based on similarity index,^6^ top 20 T‐cell clonotypes correlated better within thyroids and blood than top 21‐1000 T‐cell clonotypes (Figure 1C). These results indicated that blood‐derived T‐cell repertoires may reflect tissue‐specific immune response and facilitate disease surveillance.

**FIGURE 1:**
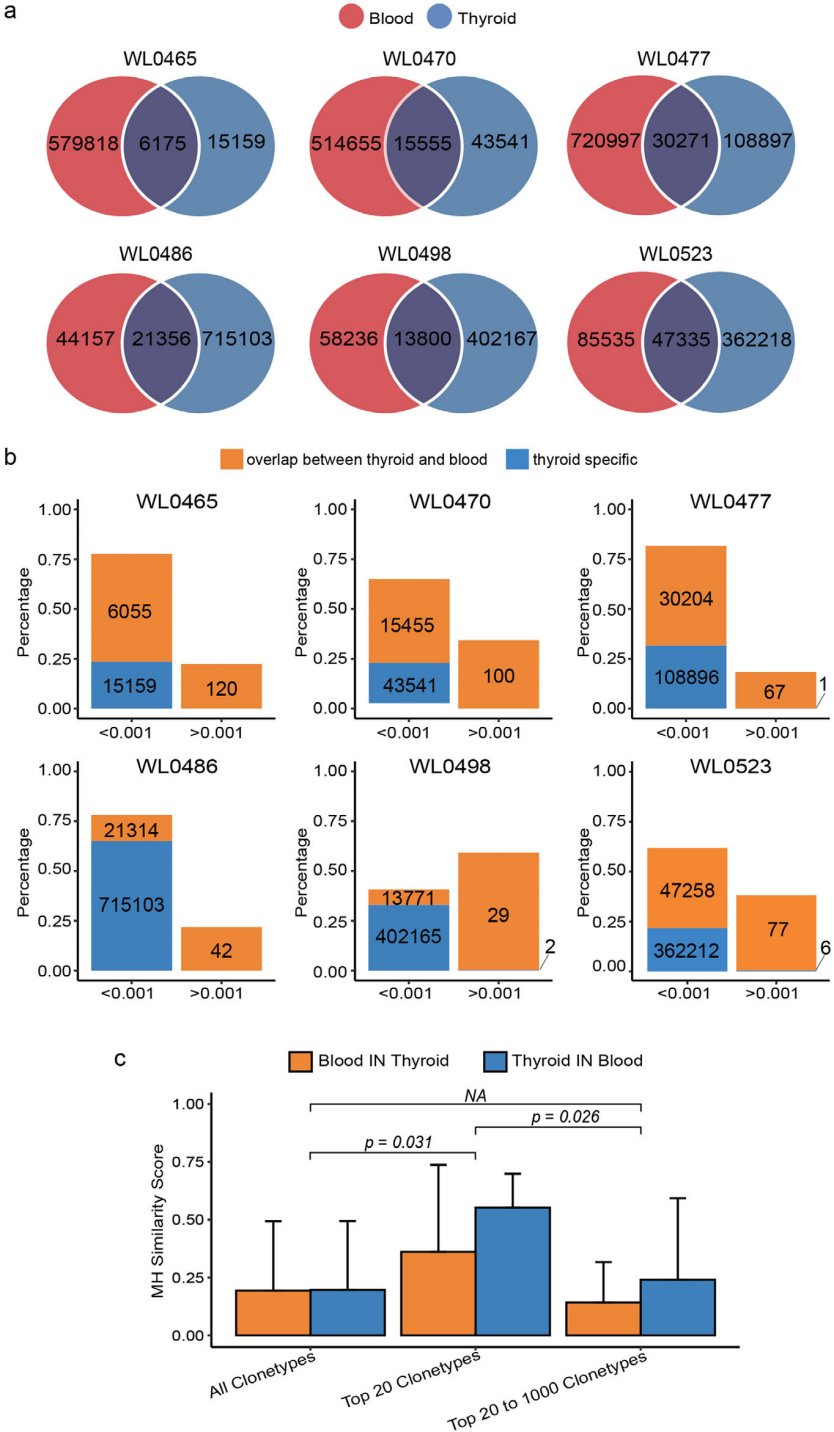
T cells showing clonal expansion in thyroids can be detected in blood. A, Overlap of T‐cell clonotypes between thyroids and blood in six GH patients. The numbers of unique clonotypes in thyroids and blood are shown in the Venn diagram. B, Cumulative frequency distribution of T‐cell clonotypes in thyroid (*y*‐axis) with frequencies below or over 0.001 (*x*‐axis). Orange bar represents the percentage of overlapped clonotypes between thyroids and blood, blue bar represents the percentage of thyroid‐specific clonotypes, and the number in the bar indicated the clonotype counts. For sample WL0465, in group of below 0.001, the percentage of overlapped clonotypes (6055) was 54.11%, and the thyroid‐specific clonotypes (15 159) was 23.55%. Whereas, in group of over 0.001, all clonotypes (120) were overlapped, and the percentage was 22.34%. C, Comparison of Morisita‐Horn (MH) similarity index between thyroids and blood at both directions in three groups including “all clonotypes,” “top 20 clonotypes,” and “top 21‐1000 clonotypes.” The orange and blue bars represent MH similarity scores of blood in thyroid and thyroid in blood in six GH patients, respectively. The range of MH similarity score is from 0 (no similarity) to 1 (absolute similarity). Error bars represent the standard deviation of MH similarity scores in each sample group. Independent two‐tailed *t*‐tests were performed to compare MH similarity scores

To further characterize specific TCR signatures, we sequenced the RNA transcripts from the complementarity determining region 3 (CDR3) of TCR Vβ from 100 peripheral blood samples, including 43 GO and 57 stable GH (Table S1). Stable GH was defined as no GO progression within 18 months follow up.^1^ All nucleotide sequences were aligned to reference TCR Vβ/Dβ/Jβ gene segments (Figure S1), and the number of reads generated was 16.277 ± 6.732 million from each sample (Table S1).

Previous studies on TCR mainly focused on the highly expanded clonotypes.^7^ Here, we represented clonal expansion by F50, which was defined as the frequency of a CDR3 sequence whose cumulative percentage reached 50% in descending‐ordered VJ (Figure 2A‐C, Figure S2, Equation S1 in the Supporting Information). And F50, rather than the total frequency could precisely depict the clonal expansion status in VJs among individuals (Figure 2A‐C). For instance, TRBV27‐TRBJ1‐5 and TRBV28‐TRBJ2‐7 were with parallel frequency of 0.063 and 0.067 in sample YL2713 and WL1046, respectively. However, their distribution of cumulative frequency differed dramatically. Specifically, there is a significant oligoclonal expansion in TRBV27‐TRBJ1‐5, whereas no such phenomenon is observed in TRBV28‐TRBJ2‐7. And F50 in TRBV27‐TRBJ1‐5 and TRBV28‐TRBJ2‐7 were 0.057 and 8.60E‐06, respectively (Figure 2B,C).

**FIGURE 2:**
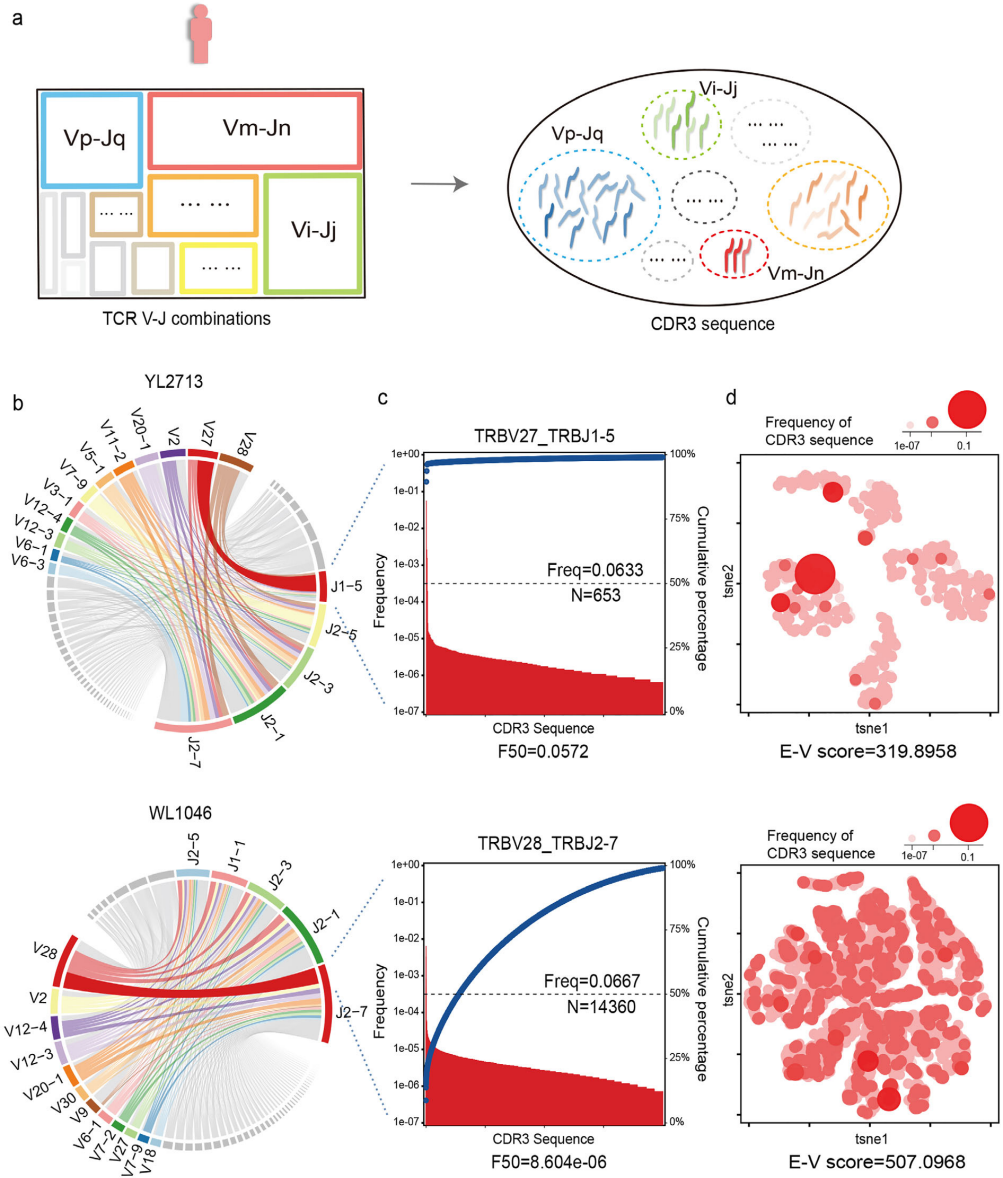
Characterization of the clonal expansion and CDR3 sequence similarity in TCR VJ combinations. A, Schematic diagram of VJ and the corresponding CDR3 sequences in individual samples. Variable number of CDR3 sequence in VJs, and for each VJ, the CDR3 sequences are heterogeneous. B, The usages of V and J genes in YL2713 and WL1046 samples are illustrated by Circos plots. The V and J genes are clockwise arranged in the order of their frequencies from low to high. VJs are illustrated by colored curved paths whose thickness represents their total frequencies in TCR repertoires. C, Example of the CDR3 sequence clonal expansion by Pareto chart on TRBV27‐TRBJ1‐5 and TRBV28‐TRBJ2‐7 in sample YL2713 and WL1046. The frequency of CDR3 sequence is represented in a descending order by red bars, and the cumulative percentages are represented by the blue point. The left and right *y*‐axes are the frequency and cumulative percentage of CDR3 sequence, respectively. The black dash line represents the F50, which was defined as the frequency of a CDR3 sequence whose cumulative frequency was 50% in VJ. In total, there were 653 and 14360 CDR3 sequences in TRBV27‐TRBJ1‐5 and TRBV28‐TRBJ2‐7, respectively. D, Example of the CDR3 sequence similarity in TRBV27‐TRBJ1‐5 and TRBV28‐TRBJ2‐7 in sample YL2713 and WL1046. t‐SNE was applied on TCRdist matrix to indicate the similarities of each CDR3 sequence pair. Each red dot represents a CDR3 sequence, and the size and color gradient indicate its frequency. Entropy‐variability (E‐V) score of TCRdist matrix represents the chaos. In this example, the E‐V score is 319.896 and 507.097 in TRBV27‐TRBJ1‐5 and TRBV28‐TRBJ2‐7, respectively

Apart from clonal expansion, it also has been suggested that the homology of CDR3 sequence plays a vital role in antigen specificity.^8^ To describe chaos of CDR3 sequence similarity, we first constructed a TCRdist matrix by measuring weighted Hamming distances of every two CDR3 sequence in VJ.^8^ And then entropy‐variability (E‐V) scores of TCRdist matrix were applied to quantify the chaos of CDR3 sequence similarity.^9^, ^10^ E and V represented the Shannon entropy and the number of different distances observed in TCRdist matrix, respectively (Figure 2D, Figure S3, Equation S2 in the Supporting Information).

It is known that T‐cell clonal expansion occurs when antigen‐driven activated and CDR3 sequence recognizing the same pMHC epitope often share conserved sequence features.^8^ Thus, we argued that when T cells are activated by specific antigens, the F50 of specific VJ would increase, and/or its corresponding chaos would decrease. Thereby, we proposed a novel score termed TCR clonal expansion and chaos score (TCS) as the ratio between F50 and chaos to characterize VJ (Figure S4, and Equation S3 in the Supporting Information).

We computed the TCS values of 100 samples (Supporting Information File S1), and selected candidate features with the best performance of classification from a training set of 33 GO and 37 stable GH (Figure S5a,b). Briefly, ‘Welch's *t*‐statistics method was used for shrinking the number of features from 514 to 75 VJs, and random forest (v4.6‐14, ntree = 1000) method was further applied to reduce the number to 13 VJs (Supporting Information File S2, Figure S5c). A diagnostic model was constructed based on the selected 13 VJs using naïve Bayes (R package, e1071: naïveBayes, v1.7‐2) classifier. The leave‐one‐out cross‐validation showed a sensitivity of 87.88% and a specificity of 81.08% to differentiate GO from stable GH (AUC = 0.817, Figure 3A, Table S2). We subsequently tested 10 GO and 20 stable GH using our diagnostic model, which achieved 80% sensitivity and 75% specificity to differentiate GO from stable GH (AUC = 0.800, Figure 3A, Table S3). To further confirm the selected features and incorporate the internal difference between GO and GH, unsupervised hierarchical clustering were performed on both training and testing dataset (Figure 3B,C). The results indicated that GO and GH can be distinguished clearly.

**FIGURE 3:**
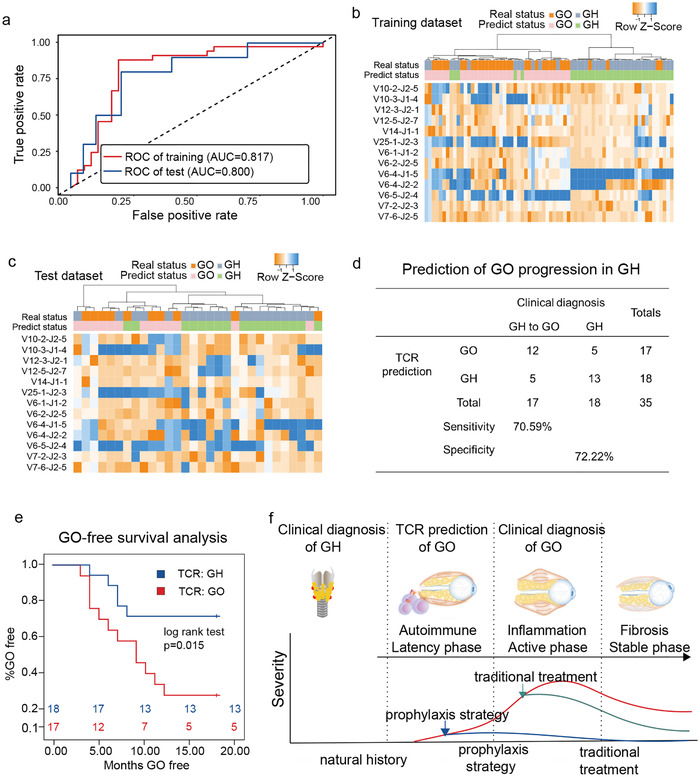
VJ signatures with potential of distinguishing GO from GH is able to predict GO progression. A, Receiver operating characteristic curve (ROC) of the diagnostic model based on 13 selected VJ signatures in the training and test datasets. B and C, The hierarchical clustering of 13 VJ signatures selected for use in the diagnostic model in the training and test datasets. D, Confusion table of binary results of 13 VJ signatures in prediction of GO progression in GH patients. E, GO‐free survival curve shows the progression of GO in initial GH patients with TCR prediction as GO or GH. F, Schematic diagram of disease progression from GH to GO. Transition from GH to GO involves a latency phase, and TCR could be applied as prospective signatures for GO progression. Prophylaxis in latency phase may be applied based on prediction of risk in GH patients

Next, we were curious about the predictive power of TCR repertoires for GO progression. Our model was applied in an independent validation study consisting of 18 GH samples and 17 “GH to GO” samples. “GH to GO” referred to initially diagnosed as GH but progressed to GO by follow up. “GH to GO” were regarded as GO in this test, and it achieved a sensitivity and specificity of 70.59% and 72.22% for prediction, respectively (Figure 3D). Specifically, 12 of the 17 “GH to GO” patients who were predicted as GO developed symptoms of eyelid swelling, chemosis, or proptosis within follow‐up period, and were confirmed of GO by computed tomography of the orbit in a median of 6.5 months (4‐9.75 months IQR, Table S4). The patients in the validation set were divided into two groups based on the prediction type, and GO‐free survival analysis was performed. The results demonstrated that “predicted as GO” patients (n = 17) were significantly different from “predicted as GH” patients (n = 18) in GO progression (*P* = .015, log‐rank test; Figure 3E). These results suggested that transition from GH to GO involves a latency phase, and TCR were prospective signatures for GO progression.

In summary, our study presented a novel score TCS combining clonal expansion of VJ and chaos of CDR3 sequence similarity. And then we developed a model for prediction of GO progression in a GH cohort as early as a median of 6.5 months, with a sensitivity and specificity of 70.59% and 72.22%, respectively. This finding suggests that GO is characterized as a spectrum of autoimmune‐mediated inflammation states, and transition from GH to GO involves a latent phase during which pathogenic TCR evolves before ophthalmopathy presents (Figure 3F). Identification of complication risk in GH patients is unprecedented, and this may open the possibility for prophylactic intervention to prevent GO. The novel approaches developed in this study may improve early detection and foster prophylactic strategy in other autoimmune diseases.

## CONFLICT OF INTEREST

The authors declare that there is no conflict of interest.

## AUTHOR CONTRIBUTIONS

Yue Wang, Bingyin Shi, Kai Ye, and Yufeng Liu conceived and designed the study. Bingyin Shi, Hui Guo, Jinkui Yang, Xiaomei Liu, Q. M., and Jingya Wang screened participants for study entry. Yufeng Liu, Xiaofei Yang, Jiadong Lin, and T. W. processed the data. Tingting Shi, Mingqian He, P. C., S. H., M. Z., and X. D. collected samples and data, and provided clinical care for participants during follow up. Liping Wu and P. H. analyzed the data with advice. Yue Wang, Kai Ye, Xiaofei Yang, and Chengsheng Zhang wrote the manuscript. All the authors contributed to the critical revision of the manuscript and approved the final version.

## Supporting information

Supporting Information

Supporting Information

Supporting Information

Supporting Information

Supporting Information

Supporting Information

Supporting Information

Supporting Information
