# Supplementary material for: Predicting the early risk of ophthalmopathy in Graves’ disease patients using TCR repertoire
Source: Clin Transl Med. 2020 Nov 4;10(7):e218. doi: 10.1002/ctm2.218 (PMC7641174; doi:10.1002/ctm2.218)
Supplement: Supplementary file 1 — Supporting Information [file CTM2-10-e218-s001.docx]

**Supplementary Materials**

**Methods:**

**Study Design.** This study was approved by the Ethic Committee of the First Affiliated Hospital of Xi’an Jiaotong University (KYLLSL-2015-004-01). Informed consent was sought from all patients before screening and study entry. Recruitment and follow-up visit for this study took place from January 2013 to December 2018 in the First Affiliated Hospital of Xi’an Jiaotong University and Beijing Tongren Hospital, Capital Medical University. We first enrolled 100 patients including 43 GO and 57 stable GH to explore the VJ signatures that could distinguish GO from GH. Stable GH was defined as no progression of GO within 18 months follow-up. Then, we enrolled an independent validation cohort consisting of 18 GH samples and 17 ‘GH to GO’ samples. ‘GH to GO’ referred to initially diagnosed as GH but progressed to GO by follow-up. The peripheral blood samples of GH, GO and ‘GH to GO’ patients were collected when they were initially diagnosed (before treatment). All diagnosis were based on clinical symptoms, serum indexes of thyroxine, TRAb, thyroid ultrasound imaging and computed tomography scanning of orbit. All GH patients were prescribed a daily starting dose of 30 mg methimazole (MMI), and followed up by the titration regimen. GO patients received high-dose intravenously methylprednisolone therapy (IVMP therapy). No statistical methods were used to predetermine sample size. Investigators were not blinded to sample group allocation.

**Inclusion criteria**. GO: 1. Moderately to severely active GO; 2. Clinical Activity Score (CAS) ≥ 3, CAS is based on the classical signs of acute inflammation (pain, redness, swelling, and impaired function) was proposed as a clinical classification to discriminate easily between the active and quiescent stages of the disease; 3. The grade of NOSPECS ≥ 4. NOSPECS classification grades exclusively for clinical severity and does not provide a means of distinguishing inflammatory progressive. Class 4 refers to extraocular muscle involvement (usually with diplopia); 4. The duration of GO is less than 3 months; 5. The age in the range of [18, 65]. GH: 1. Newly diagnosed (duration < 2 months); 2. The age in the range of [18, 65].

**Exclusion criteria**. GO: 1. Patients who were infected by fungus, mycobacterium tuberculosis, hepatitis virus and/or any other uncontrolled bacterium, virus and fungus; 2. Patients who are allergic to methylprednisolone; 3. Pregnant and lactating women; 4. Combined with other autoimmune diseases. 5. Patients who are suffering from active peptic ulcer, recent gastrointestinal surgery, serious diabetes, hypertension, hyperlipidemia, glaucoma, osteoporosis, heart disease, cardiac failure, hepatic impairment, impaired renal function and urolithiasis; GH: 1. Patients who were infected by fungus, mycobacterium tuberculosis, hepatitis virus and/or any other uncontrolled bacterium, virus and fungus; 2. Patients who are allergic to anti-thyroid drugs; 3. Pregnant and lactating women; 4. Combined with other autoimmune diseases; 5. Patients who are suffering from one or two manifestations, including pretibial myxedema, heart disease and hepatic impairment.

**TCR sequencing and sequence analysis.** We sequenced the RNA transcripts from the complementarity determining region 3 (CDR3) of TCR Vβ from 135 samples, including 43 GO, 75 GH and 17 ‘GH to GO’ patients. PBMCs were isolated by Ficoll-Paque (GE Healthcare) density gradient centrifugation from 10ml fresh peripheral blood samples. Total RNA was isolated using Trizol reagent (Invitrogen, USA), and the sample QC was done by Agilent 2100 Bio analyzer. The library construction([9](#_ENREF_9)) was as follows. Briefly, the first round of PCR amplification of TCR CDR3 using the HTBI primers (Huntsville, Alabama, America) was carried out by Qiagen OneStep RT-PCR kit, which was followed by the second round of PCR amplification using the multiplex PCR Kit (Hilden, Nordrhein-Westfalen, Germany) to construct the libraries. After size selection and purification, paired-end sequencing of samples with a read length of 100 bp were subjected to high-throughput sequencing using the Illumina Hiseq2000 platform.

Raw sequence data was converted to FASTA files using SOAPnuke (http://soap.genomics.org.cn/soapfuse.html). Reads contaminated by the adapter sequence, with uncalled bases, with a Phred quality score average below 15, or with no exact match to the TCRβ-constant primer or a multiplex identifier were discarded. Then, we merged the high-quality paired reads using software COPE (v1.2.5, developed by BGI, Shenzhen, China). We designated these unique nucleotide sequences as clonotypes. All clonotypes were aligned to reference TCR Vβ/Dβ/Jβ gene segments from the IMGT (http://www.imgt.org/) database using miTCR (v1.1, developed by MiLaboratory, http://mitcr.milaboratory.com/downloads/). In this study, we selected VJs with frequency larger than 0.0001 (99.2% of TCR repertoires) for further analysis.

**Similarity index calculation.** Similarities of TCR between blood and thyroids compartments were calculated with Morisita-Horn (MH) similarity index, comparing all unique numbers of clonotypes of one compartment with the repertoire of the other for each individual sample, and was given by

$MH similarity index= \frac{2\sum_{i=1}^{n} {x_{ij}x}_{ik}}{\left[ \left( \sum_{i=1}^{n} x_{ij}^{2}/N_{j}^{2} \right)+\left( \sum_{i=1}^{n} x_{ik}^{2}/N_{k}^{2} \right) \right]N_{j}N_{k}}$ (1)

x_ij_ and x_ik_ are the numbers of reads of a specific clonotype i in samples j (blood) and k (thyroid), N_j_ ($\sum_{i=1}^{n} x_{ij}$) is the total number of reads in sample j; and N_k_ ($\sum_{i=1}^{n} x_{ik}$) is the total number of reads in sample k.

**TCS definition.** We characterized both clonal expansion of VJ (F50) and chaos of peptide similarity. F50 was defined as the frequency of a CDR3 sequence whose cumulative percentage reached 50% in descending-ordered VJ, and was given by

$F50=f\left( x_{i} \right) s.t \sum_{0}^{i} f(x_{i})\geq0.5$, (2)

in which *x_i_* was a CDR3 sequence in descending-ordered VJs, and *f (x_i_)* was the frequency of CDR3 sequence *x_i_*. We calculated chaos of peptide similarity as E×V, where

$E=-\sum\{\left[ log Freq\left( TCR dist \right) \right]\text{×}\mathrm{Freq}\left( TCR dist \right)\}$, (3)

and V represented the number of different types observed in TCRdist matrix. The TCRdist measured distance of two peptides in the same VJ, and it was measured by comparing these peptide sequences using a weighted Hamming distance, with a gap penalty introduced to capture variation in length. Specifically, we aligned every two CDR3 sequence in VJ by Needleman-Wunsch algorithm (Saul Needleman and Christian Wunsch, 1970). And then, TCRdist was calculated based on BLOSUM62 substitution matrix (<http://www.uky.edu/Classes/BIO/520/BIO520WWW/blosum62.htm>) as follows: distance (a, a)=0; distance (a, b)=min(4,4-BLOSUM62(a, b)), where a and b were amino acids. In addition, gap opening and gap expansion penalty were both set as 8. For each VJ, we computed TCRdist of every peptide pair.

At last, we proposed a novel score termed TCS as the ratio between F50 and E×V to quantify VJ, and was given by

$TCS={\frac{-1}{log(F50)}}/{log(chaos)}$, (4)

**Statistics.** All data are means ± SD. Statistical analyses were performed by unpaired two-tailed Student’s t test. Differences were considered statistically significant at P < 0.05. Statistical evaluation of survival curves was performed using log-rank test.

**Data available.** Raw FASTQ files were uploaded to the NCBI SRA database (https://www.ncbi.nlm.nih.gov/sra) with an approval accession number SRP182582. TCS code we used is available on github (<https://github.com/xjtu-omics/TCS>).

**Figures:**

**Supplementary figure 1. T cells showing clonal expansion in thyroids can be traced in blood.** (a) Overlap of T cell clonotypes between thyroids and blood in six GH patients. (b) Frequency distribution of clonotypes in thyroids confirmed that the majority of high expanded clonotypes (above 0.1%) were overlapped with blood. (c) Comparison of all T cell clonotypes, top 20 clonotypes and top 21 to 1000 clonotypes between thyroids and blood at both directions in six GH patients. MH can range from 0 (no similarity) to 1 (absolute similarity). Error bars represent the standard deviation between samples of the same group.

**Supplementary figure 2. The** **effect of multiple sequence alignment on frequency distribution of VJ.** As the same nucleotide sequence might be aligned to different VJ combinations, we explored the effect of multiple sequence alignment on frequency distribution of VJs. (a) VJ frequency distribution histogram from all samples. Blue histogram represented frequency distribution of VJs consisting of all nucleotide sequences, whereas red represented frequency distribution of VJs consisting of the nucleotide sequences which have filtered multiple-aligned sequences. The correlation analysis between the two groups showed a strong correlation. (b-d) VJ frequency distribution histogram and correlation analysis of representative sample from GO, GH and ‘GH to GO’, respectively.

**Supplementary figure 3. Definition of F50.** (a-c) The usages of V and J genes are illustrated by circos plots. The V and J genes are clockwise arranged in the order of their frequencies from low to high. VJs are illustrated by colored curved paths whose thickness represent their Total frequencies (Ft) in TCR repertoires. (d-f) Pareto chart shows frequency distribution of CDR3 sequence within VJs. The peptides are represented in descending order by bars (red), and the cumulative percentage is represented by the line (blue). The left and right vertical axis are the frequency (-1/log freq) and cumulative percentage of CDR3 sequence, respectively. F50 is defined as the frequency of a CDR3 sequence whose cumulative frequency reaches 50% in VJs. And F50 rather than Ft could precisely depict the clonal expansion status in VJs among individuals. For instance, TRBV10-3-TRBJ2-1 and TRBV12-3-TRBJ2-5 are with parallel Ft of 0.023 and 0.024 in sample YL2386 and WL0601. However, their distribution of cumulative frequency differed dramatically. Specifically, there is a significant oligoclonal expansion in TRBV10-3-TRBJ2-1, whereas no such phenomenon is observed in TRBV12-3-TRBJ2-5. And F50 in TRBV10-3-TRBJ2-1 is 0.016, while TRBV12-3-TRBJ2-5 is 1.44E-05. (g) Kendall rank correlation analysis between F50 and Ft.

**Supplementary figure 4. Construction of TCS score.** (a-c) Chaos of peptide similarity are depicted by TCRdist matrix. TCRdist measures as a weighted Hamming distance of two CDR3 sequence in the same VJ, and t-SNE is applied to visualize the matrix of TCRdist (red dots). Each dot represents a CDR3 sequence, and its size indicates the frequency. F50 and chaos are two key features of VJs. TRBV10-3-TRBJ2-1 and TRBV15-TRBJ2-3 are with comparable F50 of 0.016 and 0.013 in sample YL2386 and WL1920. However, their TCRdist matrix differ dramatically, and t-SNE shows that there is no obvious centre in TRBV10-3-TRBJ2-1, whereas there are several clusters formed in TRBV15-TRBJ2-3. (d, e) Chaos of TCRdist matrix is quantified by entropy- variability (E-V) score (ref 17, 18). E and V represents the Shannon entropy and the number of different types observed in TCRdist matrix, respectively. The detailed calculation methods and formulas are shown. (f) The TCS score is calculated as the ratio between F50 and E-V.

**Supplementary figure 5. Kendall rank correlation analysis between every two variables in TCS score.**

**Supplementary figure 6. Generation the diagnostic model using TCR signatures.** (a) Workflow chart of TCR model establishment and leave-one-out cross validation. Briefly, the ‘Welch's t-statistics’ method were used for shrinking the variance from 514 to 75 VJs. 100 patients were randomly split into training and test set with a ratio of 7:3. Random forest and leave-one-out cross validation (LOOCV) were applied to select features with the most distinguishing power to classify GO from GH. Top 30 important features were selected as a feature set in each LOOCV process, and repeated 70 times. Following Sequential Forward Selection strategy, top 13 VJ features with the highest accuracy based on naïve Bayes were selected. As a result, a naïve Bayes classifier was built based on 13 VJ features selected above. (b) The top 13 most frequent VJs were selected into diagnostic model which provided the highest accuracy of 0.84. (c) Common percentage of 13 VJs shared in training or test samples. The horizontal axis represented 13 VJs in the order of their importance, and the vertical axis indicated the percentage that individual VJ shared either in training (blue) or test (red) samples.

**Tables:**

**Supplementary Table 1 | Patient information and sequencing data**

| **patient category** | **patient ID** | **Initial clinical diagnosis** | **TCR diagnosis** | **Clinical information** | | | | | **TCR sequencing** | | |
| --- | --- | --- | --- | --- | --- | --- | --- | --- | --- | --- | --- |
|  |  |  |  | **Gender** | **Age** | **FT4 (pmol/L)** | **CAS** | **Therapy** | **Clean Reads** | **Unique clonotypes (unique nucleotide sequences)** | **Cdr3**  **sequence** |
| GO | YL2620 | GO | GO | F | 31 | 84.19 | 4 | IVMP | 17232823 | 241665 | 215659 |
|  | YL2622 | GO | GH | F | 35 | 34.7 | 5 | IVMP | 17655174 | 280422 | 255355 |
|  | YL2561 | GO | GO | M | 56 | 10.8 | 4 | IVMP | 19361897 | 105245 | 98102 |
|  | YL2563 | GO | GO | F | 42 | 15.1 | 3 | IVMP | 20093195 | 71571 | 67058 |
|  | YL2567 | GO | GO | F | 41 | 150.2 | 3 | IVMP | 21708996 | 334375 | 295813 |
|  | YL2572 | GO | GO | M | 32 | 19.9 | 3 | IVMP | 21694511 | 68524 | 64549 |
|  | YL2578 | GO | GO | F | 18 | 41.2 | 4 | IVMP | 23585940 | 231885 | 212960 |
|  | YL2585 | GO | GO | F | 42 | 106 | 5 | IVMP | 24874363 | 124143 | 115567 |
|  | YL2593 | GO | GO | F | 43 | 79.3 | 3 | IVMP | 26423236 | 191650 | 176976 |
|  | YL2602 | GO | GO | F | 50 | 10.8 | 3 | IVMP | 14700815 | 103652 | 97441 |
|  | YL2603 | GO | GO | M | 51 | 13 | 3 | IVMP | 18163869 | 153343 | 142953 |
|  | YL2614 | GO | GO | M | 28 | 57.23 | 6 | IVMP | 22126651 | 188583 | 175008 |
|  | YL2318 | GO | GO | F | 38 | 21.28 | 5 | IVMP | 25658666 | 19249 | 17034 |
|  | YL2333 | GO | GO | F | 52 | 14.98 | 3 | IVMP | 21247048 | 29103 | 26126 |
|  | YL2358 | GO | GO | M | 49 | 17.81 | 6 | IVMP | 21914917 | 20052 | 18253 |
|  | YL2386 | GO | GO | M | 47 | 22.55 | 5 | IVMP | 22246640 | 22902 | 20862 |
|  | YL2417 | GO | GO | F | 36 | 39 | 4 | IVMP | 36797011 | 30662 | 27699 |
|  | YL2560 | GO | GO | F | 60 | 16.03 | 4 | IVMP | 30599531 | 29974 | 26732 |
|  | YL2451 | GO | GO | F | 34 | 51.04 | 4 | IVMP | 26924203 | 32790 | 28695 |
|  | YL2486 | GO | GO | M | 45 | 10 | 3 | IVMP | 29363752 | 21184 | 18691 |
|  | YL2524 | GO | GO | F | 41 | 15.8 | 6 | IVMP | 26234514 | 20448 | 16636 |
|  | YL2528 | GO | GO | F | 33 | 59 | 4 | IVMP | 28748203 | 7897 | 6174 |
|  | YL2623 | GO | GO | F | 51 | 7.97 | 3 | IVMP | 15542545 | 458296 | 414351 |
|  | YL2625 | GO | GO | M | 54 |  | 3 | MMI | 10727958 | 169897 | 156120 |
|  | YL2628 | GO | GO | M | 29 | 17.5 | 3 | IVMP | 9885713 | 293276 | 269431 |
|  | YL2632 | GO | GH | F | 40 | 10.9 | 3 | MMI | 9528481 | 389184 | 347628 |
|  | YL2637 | GO | GO | M | 26 |  | 3 | MMI | 14948891 | 242587 | 218409 |
|  | YL2643 | GO | GO | F | 19 | 7.27 | 3 | MMI | 12945851 | 348019 | 312116 |
|  | YL2650 | GO | GO | F | 62 | 8.92 | 3 | MMI | 17607708 | 188050 | 175331 |
|  | YL2658 | GO | GO | M | 44 | 8.32 | 4 | IVMP | 13386946 | 286462 | 252841 |
|  | YL2667 | GO | GH | F | 20 |  | 3 | MMI | 21813827 | 192461 | 181342 |
|  | YL2677 | GO | GH | F | 26 | 5.4 | 3 | MMI | 16143604 | 434901 | 397281 |
|  | YL2688 | GO | GH | M | 53 |  | 3 | MMI | 11804387 | 274389 | 254675 |
|  | YL2700 | GO | GO | M | 31 | 20.6 | 3 | MMI | 9391640 | 325168 | 279129 |
|  | YL2713 | GO | GO | F | 69 | 6.46 | 4 | IVMP | 10540199 | 253881 | 233108 |
|  | YL2727 | GO | GO | M | 63 |  | 4 | IVMP | 10339886 | 289780 | 267413 |
|  | YL2742 | GO | GO | M | 53 | 93.5 | 3 | MMI | 14749454 | 248421 | 226089 |
|  | YL2758 | GO | GO | M | 60 | 6.47 | 3 | MMI | 13718640 | 237435 | 219487 |
|  | YL2775 | GO | GH | F | 35 | 16.5 | 4 | MMI | 13446752 | 322866 | 292969 |
|  | YL2793 | GO | GO | M | 30 | 12.3 | 4 | MMI | 9592025 | 126656 | 115658 |
|  | YL2812 | GO | GO | F | 77 | 10.9 | 3 | MMI | 11604070 | 216912 | 188631 |
|  | YL2832 | GO | GO | F | 34 | 29.7 | 3 | MMI | 3783206 | 282159 | 264402 |
|  | YL2853 | GO | GO | F | 48 |  | 3 | MMI | 6177591 | 228320 | 213948 |
| GH | WL0465 | GH | GH | M | 54 | 39.9 | 0 | MMI | 10044112 | 274165 | 241942 |
|  | WL0470 | GH | GH | F | 24 | 48.3 | 0 | MMI | 14110955 | 230354 | 204370 |
|  | WL0477 | GH | GH | F | 40 | 111.1 | 0 | MMI | 17374823 | 357028 | 302853 |
|  | WL0486 | GH | GO | M | 28 | 97.3 | 0 | MMI | 16908288 | 21173 | 17991 |
|  | WL0487 | GH | GO | F | 40 | 50.4 | 0 | MMI | 15494666 | 44026 | 36738 |
|  | WL0498 | GH | GO | F | 59 | 70.58 | 0 | MMI | 20651770 | 22963 | 19189 |
|  | WL0510 | GH | GO | F | 33 | 29.1 | 0 | MMI | 17133819 | 19689 | 16378 |
|  | WL0523 | GH | GH | F | 32 | 75.31 | 0 | MMI | 16081685 | 55762 | 50201 |
|  | WL0537 | GH | GH | M | 28 | 40.12 | 0 | MMI | 12038215 | 399137 | 316379 |
|  | WL0597 | GH | GH | F | 23 | 149.8 | 0 | MMI | 16901487 | 209227 | 192096 |
|  | WL0601 | GH | GH | M | 24 | 42.4 | 0 | MMI | 19062407 | 253846 | 226312 |
|  | WL0606 | GH | GH | F | 25 | 38.25 | 0 | MMI | 15511642 | 287998 | 256713 |
|  | WL0621 | GH | GO | F | 29 | 25.6 | 0 | MMI | 12266127 | 197978 | 182455 |
|  | WL0622 | GH | GO | F | 28 | 80.8 | 0 | MMI | 12538674 | 212988 | 195175 |
|  | WL0634 | GH | GO | F | 32 | 53.4 | 0 | MMI | 15017700 | 204620 | 189909 |
|  | WL0542 | GH | GO | F | 47 | 12.79 | 0 | MMI | 24625317 | 6125 | 5696 |
|  | WL0546 | GH | GO | F | 39 | 9.01 | 0 | MMI | 27461290 | 16206 | 14458 |
|  | WL0557 | GH | GO | F | 30 | 19.34 | 0 | MMI | 34222296 | 14204 | 12439 |
|  | WL0564 | GH | GO | M | 58 | 13.8 | 0 | MMI | 31349465 | 24730 | 21898 |
|  | WL0572 | GH | GH | M | 48 | 26.49 | 0 | MMI | 27217672 | 58068 | 51956 |
|  | WL0650 | GH | GO | M | 54 | 39.9 | 0 | MMI | 13529130 | 317696 | 254570 |
|  | WL0695 | GH | GH | F | 24 | 40 | 0 | MMI | 13279853 | 585814 | 482283 |
|  | WL0690 | GH | GO | F | 40 | 111.1 | 0 | MMI | 19627468 | 187127 | 159029 |
|  | WL0659 | GH | GH | M | 28 | 97.3 | 0 | MMI | 30838092 | 249336 | 213430 |
|  | WL0670 | GH | GH | F | 59 | 70.58 | 0 | MMI | 31768574 | 330694 | 276901 |
|  | WL0683 | GH | GH | F | 32 | 75.31 | 0 | MMI | 31220953 | 203470 | 162204 |
|  | WL0696 | GH | GH | F | 28 |  | 0 | MMI | 9639775 | 241570 | 226461 |
|  | WL0698 | GH | GH | F | 63 | 59.3 | 0 | MMI | 12560169 | 275419 | 253303 |
|  | WL0701 | GH | GH | F | 32 |  | 0 | MMI | 6530094 | 384324 | 346130 |
|  | WL0705 | GH | GH | M | 35 | >200 | 0 | MMI | 7982862 | 391283 | 357801 |
|  | WL0710 | GH | GH | F | 45 | 42.7 | 0 | MMI | 15928187 | 398925 | 358382 |
|  | WL0716 | GH | GO | F | 20 | 19.4 | 0 | MMI | 12366053 | 272065 | 254018 |
|  | WL0740 | GH | GH | F | 24 | 30.7 | 0 | MMI | 10406748 | 346577 | 312514 |
|  | WL0750 | GH | GH | F | 51 | 15.2 | 0 | MMI | 10227198 | 309027 | 282467 |
|  | WL0773 | GH | GH | F | 42 | 58.2 | 0 | MMI | 15481725 | 288187 | 268318 |
|  | WL0786 | GH | GH | F | 40 | 23.4 | 0 | MMI | 18311697 | 475803 | 433874 |
|  | WL0800 | GH | GH | F | 59 |  | 0 | MMI | 17651755 | 216093 | 198410 |
|  | WL0815 | GH | GH | F | 29 | 120.2 | 0 | MMI | 13823170 | 314980 | 290837 |
|  | WL0831 | GH | GH | F | 34 | 55.3 | 0 | MMI | 11754093 | 293734 | 271135 |
|  | WL0848 | GH | GH | F | 23 | 13 | 0 | MMI | 10747208 | 296151 | 270842 |
|  | WL0866 | GH | GH | M | 28 | 11.3 | 0 | MMI | 9894412 | 348722 | 312466 |
|  | WL0885 | GH | GH | F | 58 |  | 0 | MMI | 12315817 | 127001 | 120493 |
|  | WL0905 | GH | GO | M | 53 | 17.7 | 0 | MMI | 12035875 | 137319 | 128609 |
|  | WL0926 | GH | GH | F | 25 | 42.3 | 0 | MMI | 10252745 | 271634 | 248574 |
|  | WL0948 | GH | GH | M | 45 | 29 | 0 | MMI | 15081901 | 240542 | 221269 |
|  | WL1020 | GH | GH | F | 55 |  | 0 | MMI | 9864702 | 183481 | 166111 |
|  | WL1073 | GH | GH | F | 30 |  | 0 | MMI | 10448676 | 254385 | 233455 |
|  | WL1101 | GH | GH | F | 27 |  | 0 | MMI | 13095068 | 487526 | 436424 |
|  | WL1130 | GH | GH | F | 34 |  | 0 | MMI | 14841718 | 385045 | 355220 |
|  | WL1160 | GH | GH | F | 55 |  | 0 | MMI | 15639453 | 170703 | 161555 |
|  | WL1223 | GH | GH | F | 25 |  | 0 | MMI | 16260523 | 336473 | 309803 |
|  | WL1256 | GH | GH | F | 47 | 66.8 | 0 | MMI | 13253941 | 301600 | 277174 |
|  | WL1290 | GH | GH | M | 50 |  | 0 | MMI | 15096395 | 445361 | 396749 |
|  | WL1436 | GH | GH | F | 41 |  | 0 | MMI | 19985089 | 224647 | 210876 |
|  | WL1475 | GH | GH | F | 30 |  | 0 | MMI | 9896331 | 413182 | 365145 |
|  | WL1515 | GH | GH | F | 46 | 24.9 | 0 | MMI | 9639460 | 213788 | 200008 |
|  | WL1598 | GH | GO | M | 52 | 47.5 | 0 | MMI | 16112851 | 152988 | 133535 |
|  | WL1641 | GH | GH | F | 50 | 16.4 | 0 | MMI | 6165527 | 138581 | 131043 |
|  | WL1685 | GH | GH | M | 32 | 69.7 | 0 | MMI | 10822791 | 205730 | 189941 |
|  | WL1730 | GH | GH | F | 27 | 67.6 | 0 | MMI | 11769380 | 245398 | 226736 |
|  | WL1823 | GH | GH | F | 37 | 59.6 | 0 | MMI | 12170409 | 297266 | 275474 |
|  | WL1871 | GH | GH | F | 37 | 115.2 | 0 | MMI | 7731787 | 222431 | 207422 |
|  | WL1920 | GH | GH | F | 49 | 12.7 | 0 | MMI | 9227408 | 347867 | 308946 |
|  | WL2021 | GH | GH | F | 26 | 93.6 | 0 | MMI | 9534530 | 279207 | 257492 |
|  | WL2073 | GH | GH | F | 50 | 70.7 | 0 | MMI | 15774559 | 80955 | 77994 |
|  | WL2126 | GH | GH | F | 25 | >200 | 0 | MMI | 10597025 | 249733 | 235923 |
|  | WL2180 | GH | GH | M | 42 | 103.6 | 0 | MMI | 10687279 | 314390 | 285939 |
|  | WL2235 | GH | GH | F | 67 | 48.2 | 0 | MMI | 13196037 | 163323 | 154085 |
|  | WL2348 | GH | GH | M | 32 | 100.6 | 0 | MMI | 12159902 | 301804 | 279541 |
|  | WL2406 | GH | GH | M | 46 | 47 | 0 | MMI | 10571377 | 135742 | 127347 |
|  | WL2465 | GH | GO | F | 37 | 75.2 | 0 | MMI | 14732974 | 186233 | 175547 |
|  | WL0731 | GH | GH | F | 38 | 30.7 | 0 | MMI | 10901354 | 280634 | 257975 |
|  | WL1046 | GH | GH | M | 29 |  | 0 | MMI | 9544750 | 444441 | 387858 |
|  | WL1361 | GH | GH | F | 35 | 150.3 | 0 | MMI | 14778874 | 160751 | 152959 |
|  | WL2291 | GH | GH | F | 30 | 80.4 | 0 | MMI | 11523711 | 402046 | 363263 |
| GH to GO | WL0613 | GH | GO | F | 46 | 27.15 | 0* | MMI | 15768961 | 149292 | 137914 |
|  | WL0648 | GH | GO | M | 43 | 49.2 | 0* | MMI | 24895616 | 140107 | 130555 |
|  | WL0682 | GH | GO | F | 25 | 15 | 0* | MMI | 25582046 | 50413 | 46092 |
|  | WL0594 | GH | GO | M | 31 | 21.63 | 0* | MMI | 23943870 | 9357 | 8284 |
|  | WL0539 | GH | GO | F | 46 | 20.61 | 0* | MMI | 25656578 | 39638 | 36024 |
|  | WL0551 | GH | GO | F | 43 | 51.18 | 0* | MMI | 35947733 | 20332 | 18250 |
|  | WL0581 | GH | GO | F | 53 | 21.51 | 0* | MMI | 28744023 | 28746 | 27706 |
|  | WL0723 | GH | GO | F | 50 | 96.9 | 0* | MMI | 11067328 | 409800 | 357733 |
|  | WL0761 | GH | GO | M | 32 | 38.7 | 0* | MMI | 9527465 | 261949 | 237946 |
|  | WL0971 | GH | GO | F | 39 |  | 0* | MMI | 10406928 | 217254 | 204559 |
|  | WL0995 | GH | GH | F | 26 |  | 0* | MMI | 7667869 | 129965 | 125118 |
|  | WL1191 | GH | GH | F | 37 | 14.39 | 0* | MMI | 15966443 | 250087 | 233628 |
|  | WL1325 | GH | GO | F | 46 |  | 0* | MMI | 13986211 | 190617 | 181002 |
|  | WL1398 | GH | GH | F | 32 | 38.6 | 0* | MMI | 15447914 | 421994 | 385185 |
|  | WL1556 | GH | GH | F | 50 | 14.3 | 0* | MMI | 14079255 | 195490 | 184235 |
|  | WL1776 | GH | GH | F | 24 |  | 0* | MMI | 10331969 | 245511 | 227767 |
|  | WL1970 | GH | GO | F | 58 | 17.5 | 0* | MMI | 15339299 | 82344 | 78264 |

FT4 is free thyroxine. CAS, Clinical Activity Score is based on the classical signs of acute inflammation (pain, redness, swelling, and impaired function) was proposed as a clinical classification to discriminate easily between the active and quiescent stages of the disease. MMI, methimazole; IVMP, pulses of intravenous methylprednisolone. In this study, we totally sequenced the RNA transcripts from the complementarity determining region 3 (CDR3) of TCR Vβ from 135 peripheral blood samples, including 43 GO patients, 75 GH patients, and 17 ‘GH to GO’ patients. The blood samples of ‘GH to GO’ patients were collected at the time point when they were initially diagnosed as GH. *The CAS were evaluated before GO progression.

**Supplementary Table 2 | Confusion table of binary results of VJ signatures in training dataset**

| Training dataset | | Clinical  diagnosis | | Totals | kappa | P |
| --- | --- | --- | --- | --- | --- | --- |
|  |  | GO | Stable GH |  |  |  |
| TCR diagnosis | GO | 29 | 7 | 36 | 33.21 | 0.000 |
|  | GH | 4 | 30 | 34 |  |  |
|  | Total | 33 | 37 | 70 |  |  |
|  | Sensitivity | 87.88% |  |  |  |  |
|  | Specificity |  | 81.08% |  |  |  |

**Supplementary Table 3 | Confusion table of binary results of VJ signatures in test dataset**

| Training dataset | | Clinical  diagnosis | | Totals | kappa | P |
| --- | --- | --- | --- | --- | --- | --- |
|  |  | GO | Stable GH |  |  |  |
| TCR diagnosis | GO | 8 | 5 | 13 | 8.213 | 0.004 |
|  | GH | 2 | 15 | 17 |  |  |
|  | Total | 10 | 20 | 30 |  |  |
|  | Sensitivity | 80% |  |  |  |  |
|  | Specificity |  | 75% |  |  |  |

**Supplementary Table 4 | TCR prediction of GO progression in GH patients**

| **Patient ID** | **Initial clinical**  **diagnosis** | **TCR predictive diagnosis** | **Clinical outcome** | **Time interval between TCR prediction and GO onset (months)** | **Date of initial clinical or TCR diagnosis** | **Date of confirmation of GO onset** |
| --- | --- | --- | --- | --- | --- | --- |
| WL0613 | GH | GO | GO | 5 | 2016.7 | 2016.12 |
| WL0648 | GH | GO | GO | 4 | 2016.7 | 2016.11 |
| WL0682 | GH | GO | GO | 9 | 2013.4 | 14.1.14 |
| WL0594 | GH | GO | GO | 11 | 2013.1 | 13.12.10 |
| WL0539 | GH | GO | GO | 6 | 2013.5 | 13.11.26 |
| WL0551 | GH | GO | GO | 10 | 2013.3 | 14.1.17 |
| WL0581 | GH | GO | GO | 12 | 2013.2 | 14.2.26 |
| WL0723 | GH | GO | GO | 4 | 2017.12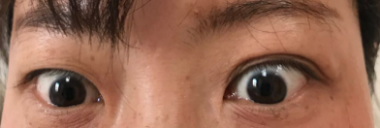 | 2018.3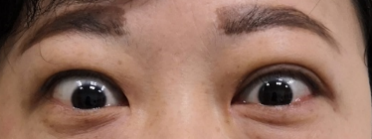 |
| WL0761 | GH | GO | GO | 9 | 2017.12  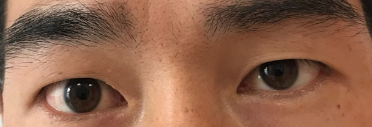 | 2018.9  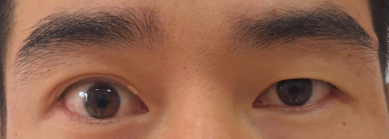 |
| WL0971 | GH | GO | GO | 7 | 2018.1.10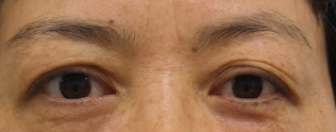 | 2018.8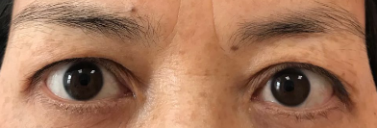 |
| WL0995 | GH | GH | GO | 7 | 2018.3.21 | 2018.10 |
| WL1191 | GH | GH | GO | 6 | 2018.3 | 2018.9 |
| WL1325 | GH | GO | GO | 3 | 2018.3 | 2018.6 |
| WL1398 | GH | GH | GO | 7 | 2018.04.11  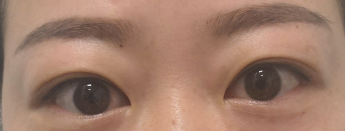 | 2018.11  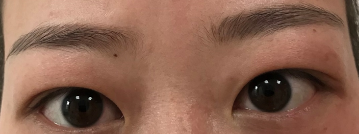 |
| WL1556 | GH | GH | GO | 8 | 2018.04.16  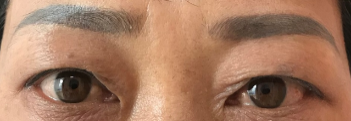 | 2018.12  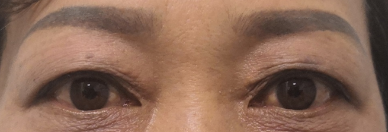 |
| WL1776 | GH | GH | GO | 4 | 2018.8.1 | 2018.12 |
| WL1970 | GH | GO | GO | 4 | 2018.4 | 2018.8 |

‘GH to GO’ refers to initially diagnosed as GH but progressed to GO by follow-up. And this model was able to predict GO progression as early as a median of 6.5 months (4-9.75, IQR).
