# Supplementary figures and images for: Predicting the early risk of ophthalmopathy in Graves’ disease patients using TCR repertoire
Source: Clin Transl Med. 2020 Nov 4;10(7):e218. doi: 10.1002/ctm2.218 (PMC7641174; doi:10.1002/ctm2.218)

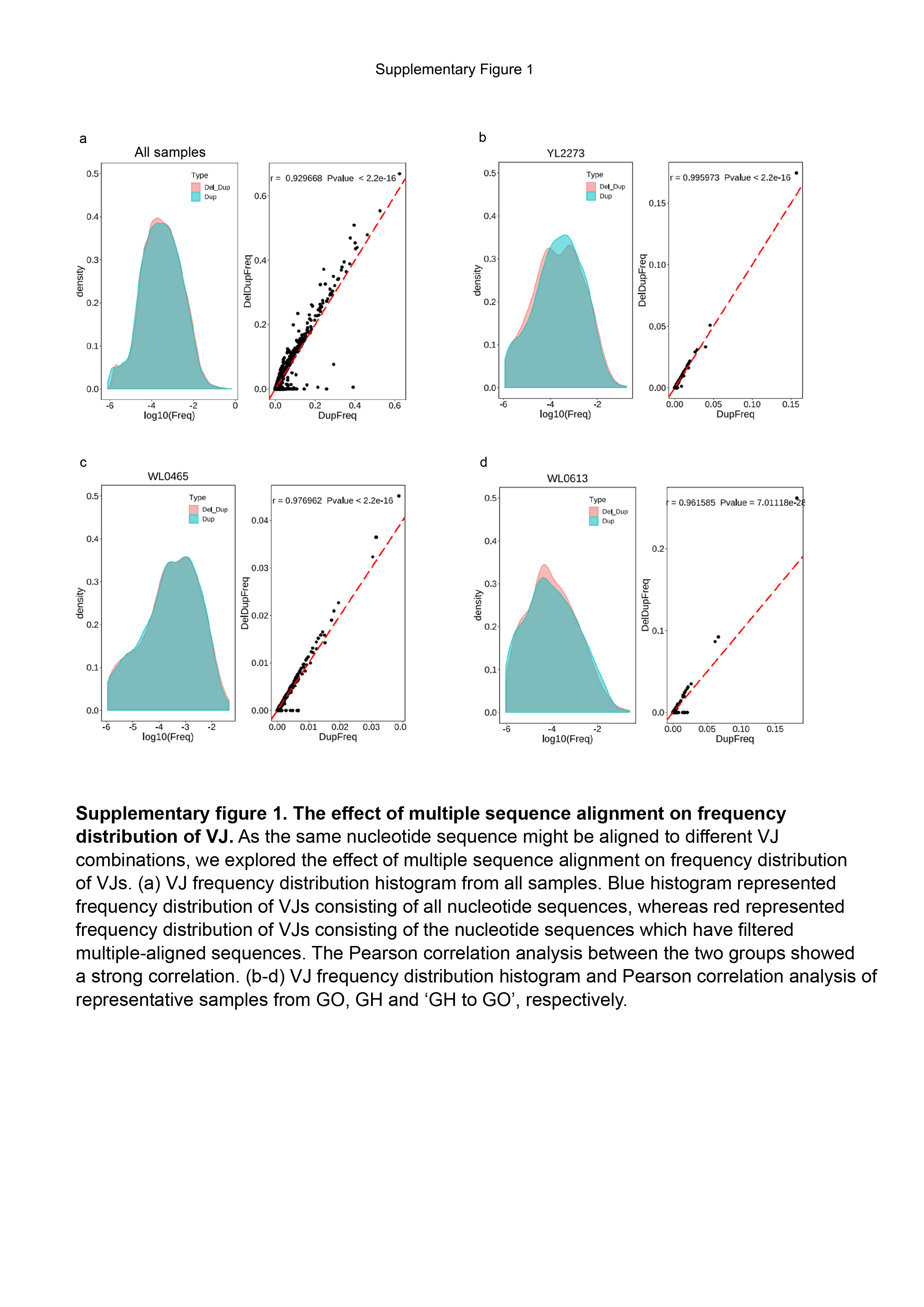

Supplement: Supplementary file 4 — Supporting Information [file CTM2-10-e218-s004.jpg]

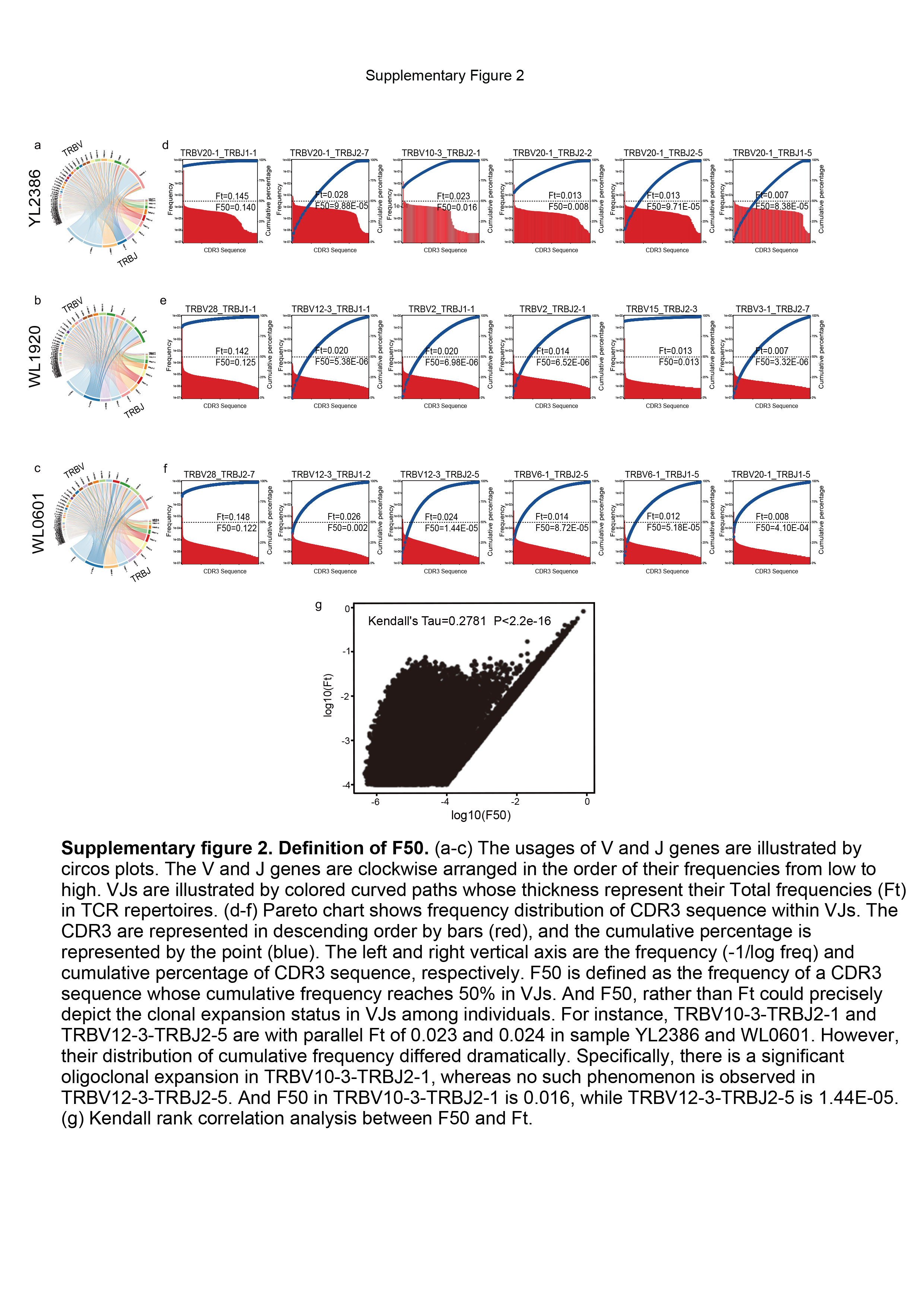

Supplement: Supplementary file 5 — Supporting Information [file CTM2-10-e218-s005.jpg]

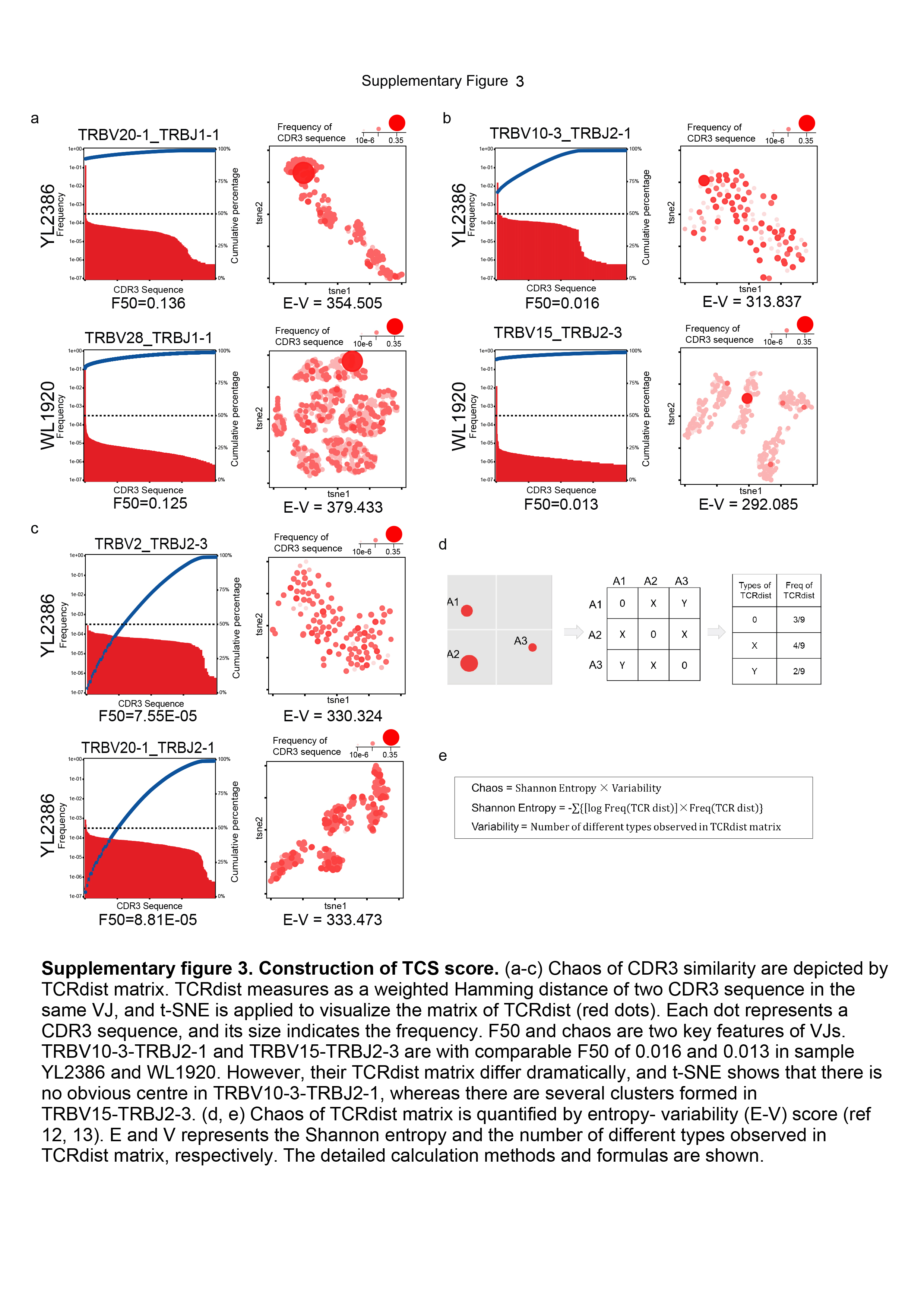

Supplement: Supplementary file 6 — Supporting Information [file CTM2-10-e218-s006.jpg]

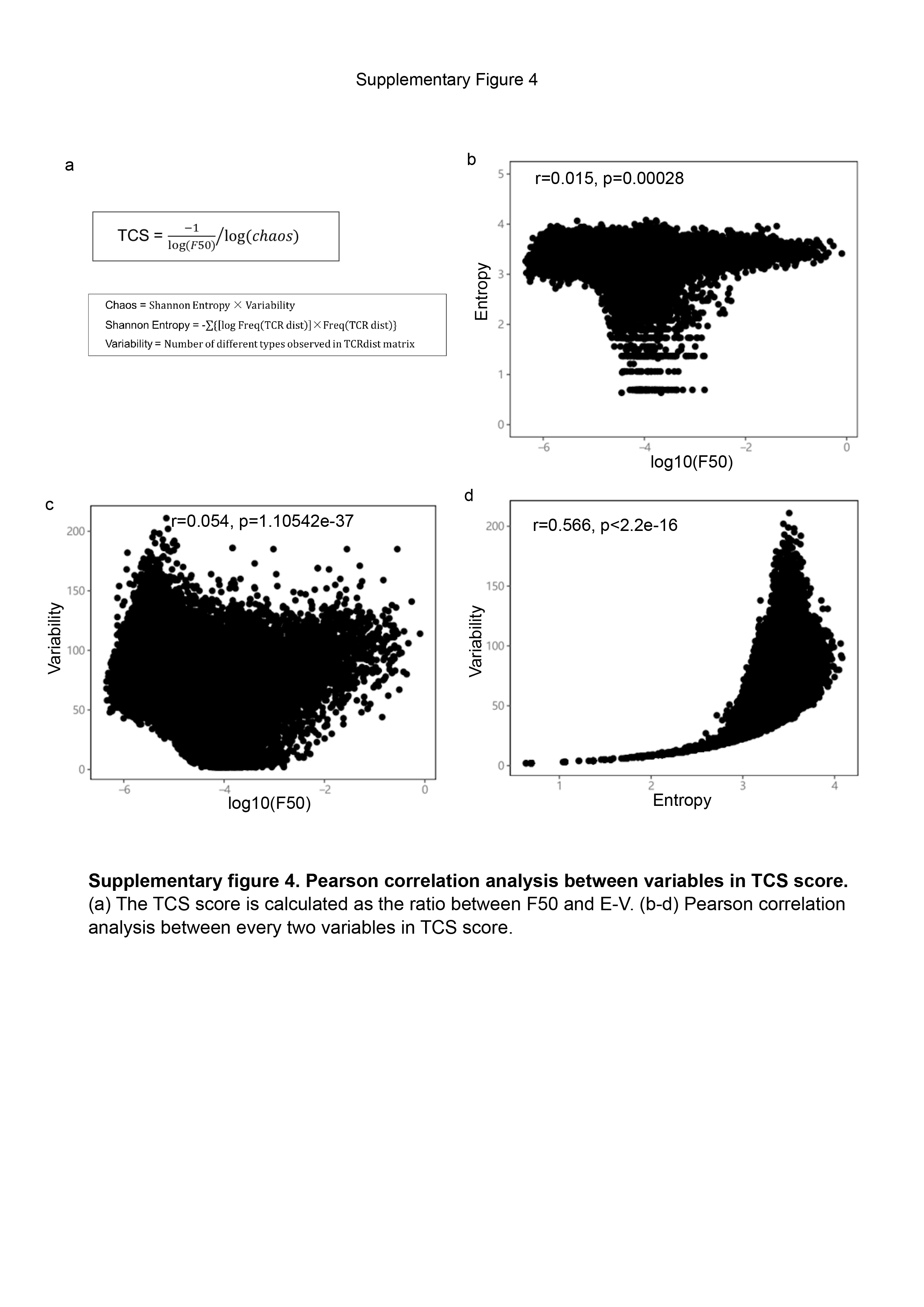

Supplement: Supplementary file 7 — Supporting Information [file CTM2-10-e218-s007.jpg]

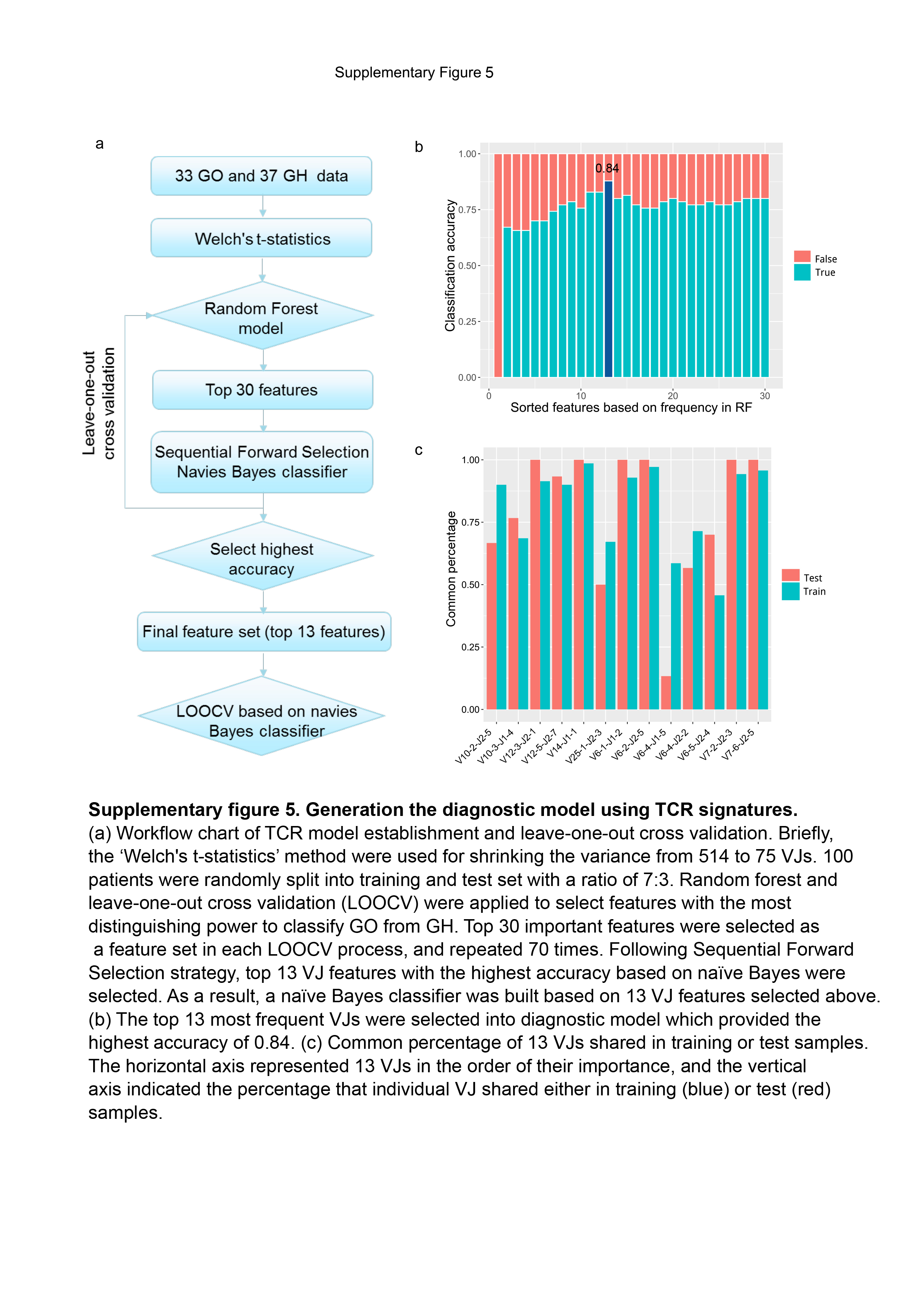

Supplement: Supplementary file 8 — Supporting Information [file CTM2-10-e218-s008.jpg]
